# Influence of Radiographic Viewing Perspective on Glenoid Inclination Measurement

**DOI:** 10.1177/2471549218824986

**Published:** 2019-06-06

**Authors:** Peter N Chalmers, Thomas Suter, Matthijs Jacxsens, Yue Zhang, Chong Zhang, Robert Z Tashjian, Heath B Henninger

**Affiliations:** 1 Department of Orthopaedic Surgery, University of Utah, Salt Lake City, Utah; 2 Department of Orthopaedic Surgery, Kantonsspital Baselland, Liestal, Switzerland; 3 Department of Orthopaedics and Traumatology, Kantonsspital St. Gallen, St. Gallen, Switzerland; 4 Division of Epidemiology, Department of Internal Medicine, University of Utah, Salt Lake City, Utah

**Keywords:** total shoulder arthroplasty, glenoid component, glenoid inclination, glenoid tilt, reliability, 3-dimensional computed tomography, digitally reconstructed radiographs, shoulder, beta angle, accuracy

## Abstract

**Introduction:**

The purposes of this study were to determine (1) whether glenoid inclination (GI) could be accurately measured on plain radiographs as compared to a gold-standard 3-dimensional (3D) measure and (2) whether GI could be reliably measured on plain radiographs.

**Materials and Methods:**

Digitally reconstructed radiographs (DRRs) were made from 3D computed tomography reconstructions of 68 normal cadaver scapulae. DRRs were made in a variety of viewing angles. Inclination was measured on these DRRs. These measurements were also made using a gold-standard 3D method. Measurements were made by 2 orthopedic surgeons and 1 surgeon twice, to calculate interrater and intrarater intraclass correlation coefficients (ICCs).

**Results:**

The gold-standard 3D *β* was 83 ± 5° (72°–98°). On neutral plain radiographs, the mean ± standard deviation 2D *β* angle was 80 ± 6° (range, 66°–99°). With regard to accuracy, the 2D *β* angle was significantly different from the 3D *β* angle, with the 2D *β* underestimating the 3D *β* by 5° (95% confidence intervals −1 to 12). With regard to reliability, interrater ICCs for 2D *β* with a neutral viewing angle was 0.79. Two-dimensional *β* varied widely with viewing angle from 0.24 to 0.88. Interrater ICCs for the 3D method was 0.83 (0.60–0.92). Intrarater ICCs for all 3 techniques were high (>0.91).

**Conclusions:**

Two-dimensional radiographic GI measurement is not accurate, as it underestimates the 3D value by an average of 5° when compared to the gold-standard 3D measurement. GI 2D measurement reliability varies with viewing angle on plain radiographs and thus to accurately and reliably measure inclination 3D imaging is necessary.

## Introduction

Abnormalities in glenoid inclination (GI) may be linked to rotator cuff tears and osteoarthritis.^1-5^ They may also be a source of failure after total shoulder arthroplasty (TSA).^6-10^ GI may thus play a role in predicting rotator cuff tears, osteoarthritis, failure after rotator cuff repair, and failure after TSA.^1,3-10^ Although an initial study suggested that GI can be accurately and reliably measured on plain radiographs,^11^ a more recent study has suggested that GI cannot be accurately measured on plain radiographs.^12^ Although 2-dimensional (2D) analyses exist,^11^ 3D computed tomographic (CT) measures are considered the gold standard.^13-20^ No studies exist comparing gold-standard 3D CT measures to radiographic measures of the native glenoid.^11,13^ Even with 3D imaging, GI cannot be accurately measured without slice reorientation into the plane of the scapula.^14^ Gold-standard 3D CT measures are complex and require specialized software capable of reslicing the voxel matrix to create a coronal image in the plane of the scale and are thus not readily applicable in a clinical setting.^13-20^ Developing a method for inclination to be reliably and accurately measured on plain radiographs is crucial for this factor to be useful clinically and for use in further research on shoulder pathology and treatment.

The reliability and accuracy of GI measurement on the anteroposterior (AP, neutral view) radiograph may depend upon the orientation of the scapula relative to the x-ray beam and cassette, which in turn depends upon the patient’s positioning and posture. The effect of scapular orientation can be studied in a controlled manner using digitally reconstructed radiographs (DRRs) generated from CT scans, a process which has previously been validated.^21^ Using DRRs, a previous study demonstrated that the critical shoulder angle (CSA), which is a compound measurement including both GI and the acromial index,^2,4,5,22,23^ could only be radiographically measured reliably and accurately on radiographs perfectly parallel to the glenoid profile.^24^ Deviation in viewing perspectives away from the neutral view was detrimental to reliable or accurate measurement of the CSA.

The purposes of this study were to determine (1) whether GI could be accurately measured on plain radiographs as compared to a gold-standard 3D measure and (2) whether GI could be reliably measured on plain radiographs. We hypothesized that the neutral (AP) view radiographs would be both accurate and reliable in measurement of GI, but radiographic viewing perspectives other than the neutral view relative to the scapula would have decreased reliability and accuracy as compared to the neutral view.

## Materials and Methods

### Data Collection

This study was performed under the University of Utah Institutional Review Board approved protocol #11755. An existing data set was utilized for this study, and the methods for cadaver selection and the creation of DRR have been described previously.^24^ These cadavers were screened for osteoarthritis and rotator cuff tears by direct visualization during dissection, and 3D CT reconstructions were evaluated by an orthopedic surgeon fellowship trained in shoulder and elbow surgery (TS). As many cadavers are advanced in age, cadavers were carefully screened for these pathologies, which are common in these age groups. Sixty-eight cadaver shoulders (25 pairs and 18 individual scapulae) were included. All cadavers underwent CT scans performed with a Siemens Sensation (Siemens Medical, Malvern, PA) CT scanner (130 kV, 512 × 512 matrix, 1.0 mm slice thickness, 0.75 pitch, 170-mAS current). These images were exported to DICOM (Digital Imaging and Communications in Medicine) format and semiautomatically segmented (Amira v5.4; Visage Imaging, San Diego, CA) and reconstructed into 3D surfaces, then used to create DRRs. The methodology for the creation of DRRs has been previously validated for reproducibility and accuracy.^21^ This sample size was selected, as it was adequate for a similar prior study, and we did not perform an a priori power analysis.^24^

### Definition of Scapular Plane and Axes

**Figure 1.**
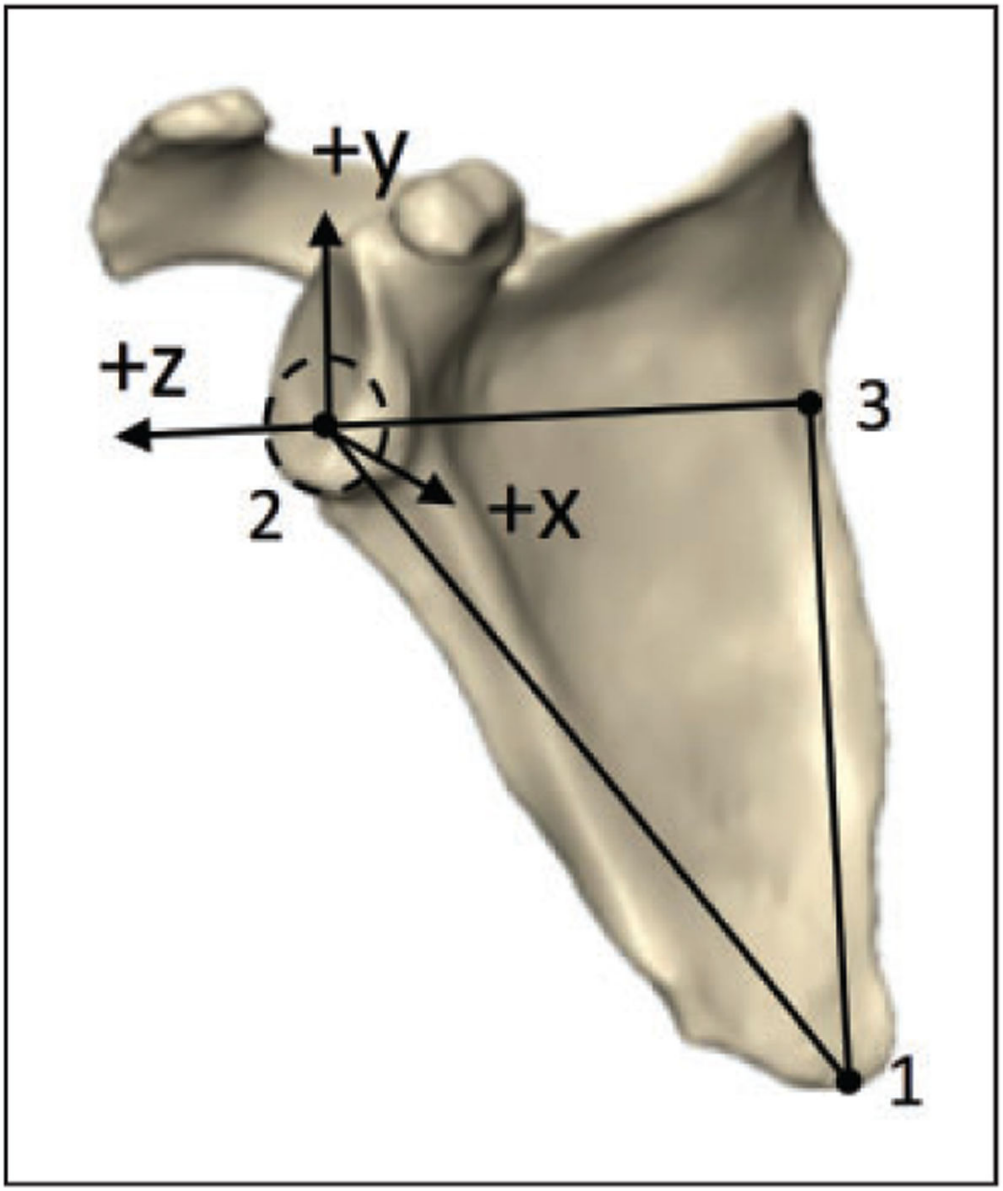
Three points were used to define the plane of the scapula on 3D CT reconstructions: *z*-axis defined as the line from the (3—trigonum) to (2—glenoid center) (+ lateral), *x*-axis perpendicular to the scapular plane defined by points (2—glenoid center)-(1—inferior angle)-(3—trigonum) (+ anterior), and the *y*-axis as the cross product of the *x*- and *z*-axes (+ superior).

On each of the 3D reconstructions, 2 independent observers determined the coordinates of the following points on the scapula: (1) the most distal point of the inferior angle, (2) the center of the glenoid (ie, the center of the best-fit circle of the inferior glenoid), and (3) the intersection between the scapular spine and the medial border (ie, the trigonum) (Figure 1). These 3 points were used to define the plane of the scapula as follows: *z*-axis defined as the line from the (3) to (2) (with the lateral direction being positive), *x*-axis perpendicular to the scapular plane defined by points (2)-(1)-(3) (with anterior direction being positive), and the *y*-axis as the cross product of the *x*- and *z*-axes (with the superior direction being positive). Left scapulae were mirrored to create right scapulae, so a right-handed coordinate system convention could be consistently applied to all models.

### DRR Generation

DRRs were then generated as previously described,^21,24^ using a ray casting technique which creates images that simulate a radiograph based on the intensity of the pixels in the CT scan and the respective viewing angle. This process has been previously validated.^21^ In each of the 68 scapulae, a neutral image was generated with a viewing perspective perpendicular to the scapular plane, corrected for glenoid version to view the glenoid in profile. In 10 randomly selected nonpaired scapulae, 20 additional viewing perspectives were created for incremental changes in anteversion, retroversion, flexion, extension, and compound rotations. These 10 scapula × 20 viewing perspectives, in addition to the 68 neutral images, provided a total of 268 DRRs. This process was not repeated for the full 68 scapulae, as doing so would have created 1428 images for analysis. The RAND function in Excel was used to assign random numbers between 0 and 1 to each specimen. The lowest 10 numbers, not part of another selected pair, were chosen for analysis to randomly select 10 scapulae.

Anteverted and retroverted viewing perspectives were created by rotation around the *y*-axis, which has also been described as internal and external rotation. Flexed and extended viewing perspectives were created by rotation around the *z*-axis, which has also been described as anterior and posterior tilting. These rotations were performed at 5°, 10°, 15°, and 30° increments in each direction. Compound rotations were created at 15° on each axis, which created a total of 20 additional viewing perspectives for each of the 10 neutral images. These images were then blinded and randomized, and each was evaluated by 3 independent board-certified orthopedic surgeons (PNC, TS, and RZT). One author also evaluated each radiograph in the neutral plane twice, separated by 2 weeks, to determine intrarater reliability.

### Protocol for 2D Inclination Measurement

**Figure 2.**
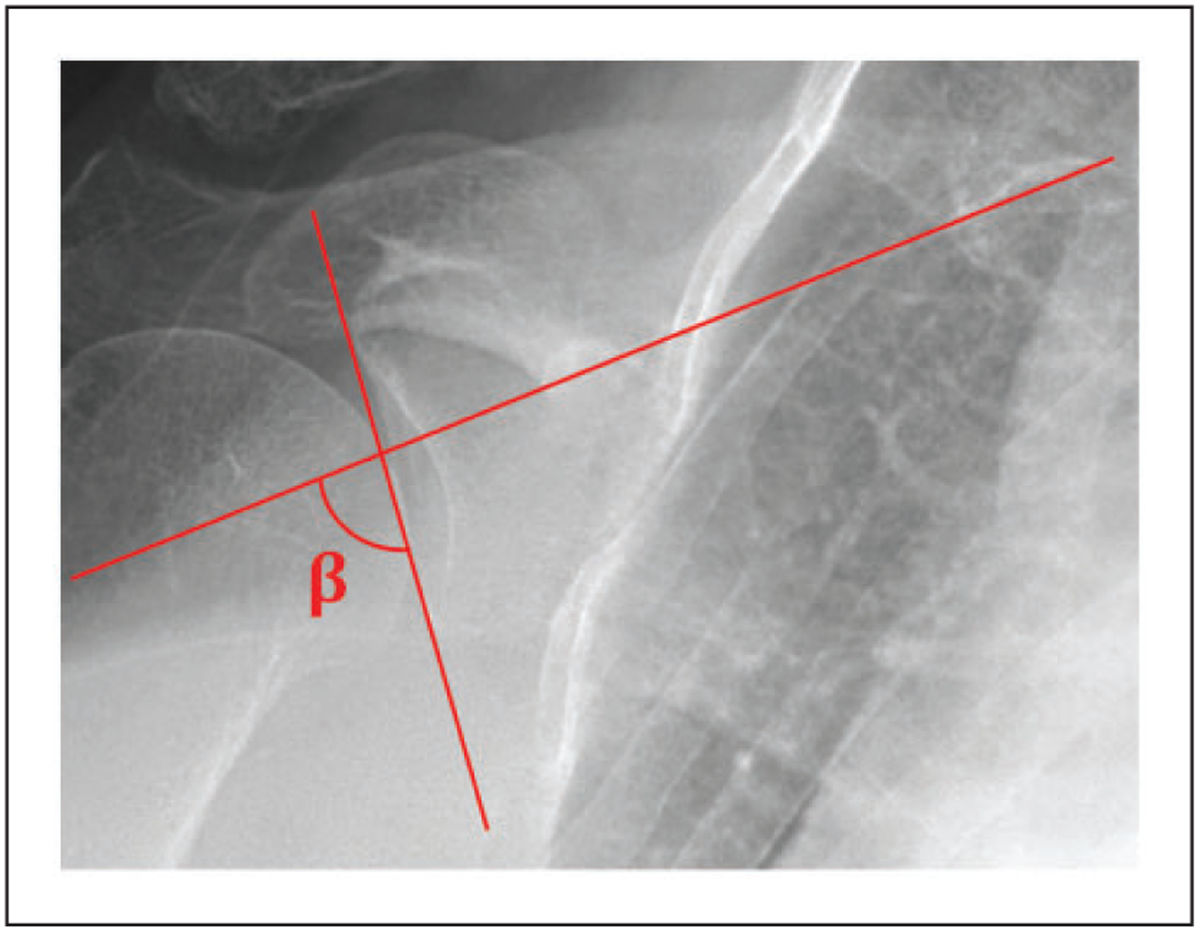
Measurement of the 2D *β* angle on a digitally reconstructed radiograph as the angle between the supraspinatus fossa (horizontal) and a line between the inferior and superior glenoid poles (vertical).

GI measurements in 2D were performed on each DRR using ImageJ (National Institutes of Health, Bethesda, MD).^25^ GI, described by Maurer et al. as the beta angle (*β*),^11^ was the angle between a line parallel to the floor of the supraspinatus fossa and a line between the superior and inferior glenoid rims (Figure 2).

### Protocol for 3D Inclination Measurement

**Figure 3.**
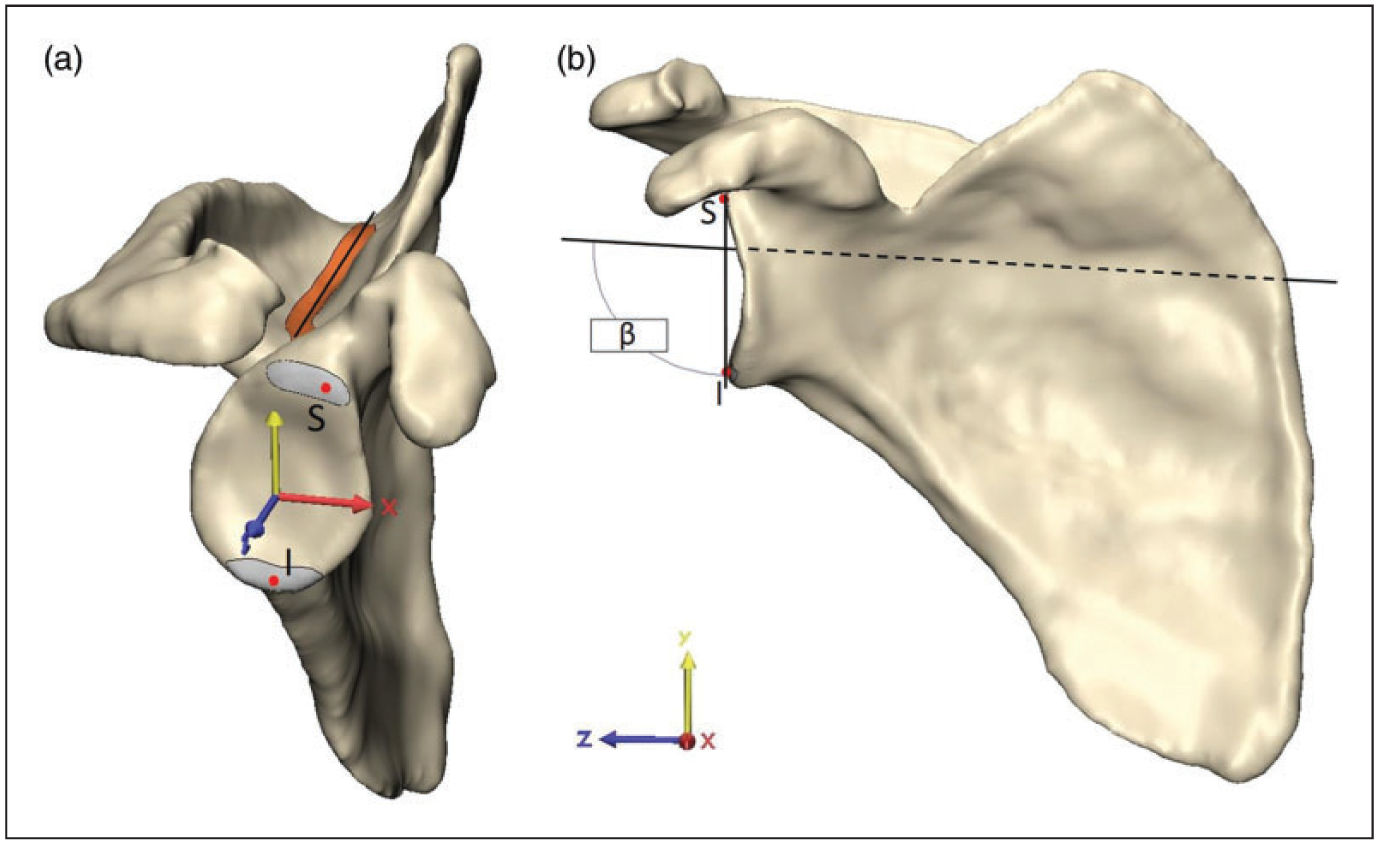
Measurement of the gold-standard 3D *β* angle. (a) The inertia axis of the floor of the supraspinatus fossa (orange, Fossa line) and most lateral points of the superior (S) and inferior pole (I) of the glenoid fossa (Glenoid poles) were acquired using built-in 3-matic functions. (b) The 3D *β* angle was then calculated between the supraspinatus fossa line and the line connecting S and I in the scapular (*yz*) plane.

The 3D method was considered the gold standard for glenoid measurement in prior scapular studies and was thus considered the gold standard in this study even though many groups do not routinely use it clinically.^13-20^ Within this study, this method is thus used for validation of radiographic methods. The aligned 3D reconstructions were imported into 3-Matic (Materialise, Leuven, Belgium). The 3D *β* angle described by Van Haver et al.^13^ was adapted to a semiautomated measuring protocol. The supraspinatus fossa line was acquired by calculating the inertia axis for the floor of the supraspinatus fossa (Figure 3(a)). The superior and inferior poles of the glenoid were independently marked over the complete glenoid width. The most lateral points of these 2 marked segments were selected with the extrema function as the most lateral point of each segment along the scapula *z*-axis (Figure 3(a)). The angle between the supraspinatus fossa line and the line connecting the most lateral point of the superior and inferior glenoid pole was then projected to the scapular (*yz*) plane providing the 3D *β* angle (Figure 3(b)).

### Statistical Analysis

Descriptive statistics were used to summarize the distribution of *β* angle measurements among the study cohort. We used linear mixed-effect models to compare the differences in *β* angle with respect to viewing perspectives. The linear mixed-effect model took in account the correlation among the repeated measurements on the same shoulder and correlation between the paired shoulders by introducing a random effect component.^26^ The intraclass correlation coefficient (ICC) was used to measure the absolute agreement in the measurement of *β* angle measurements for each viewing perspective. An ICC of ≥0.75 was used in this study as the threshold for acceptable agreement between raters, as suggested in a prior publication.^27^ In addition, paired Student’s *t* tests were performed to compare each measurement type. Bland-Altman plots were also created to visualize the differences between techniques.^28^ All statistical tests were conducted at a significance level of .05. All analyses were performed with the R statistical packages.^29^

## Results

### Accuracy of Radiographic Measurement of GI

**Figure 4.**
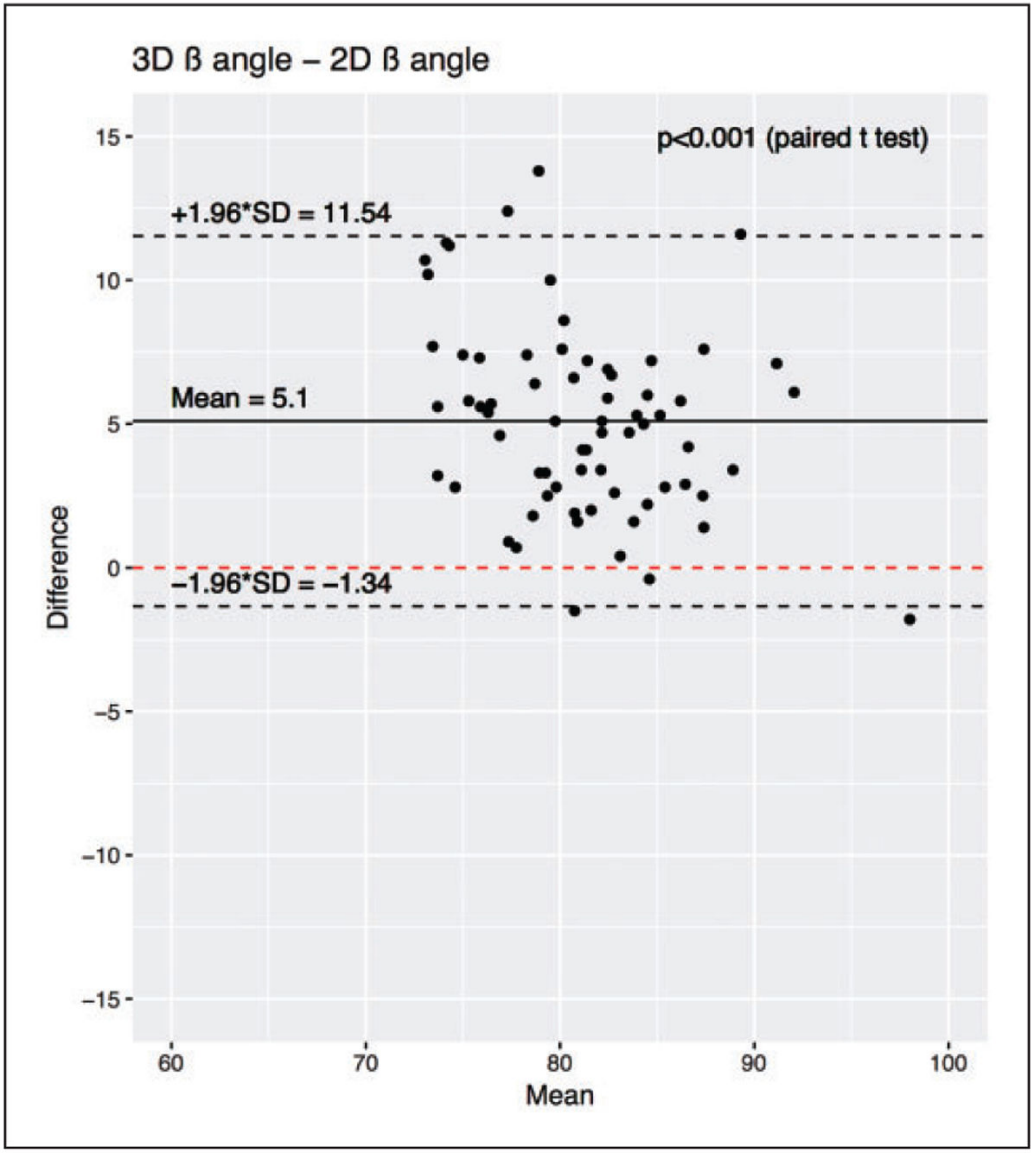
Bland-Altman plot demonstrating the difference between 2D *β* and 3D *β* angle measures. The mean difference is denoted by the solid black line, and the 95% confidence intervals of the mean difference are shown by dashed black lines. *P* values show the results of a paired Student’s *t* test between the respective populations. SD, standard deviation.

**Figure 5.**
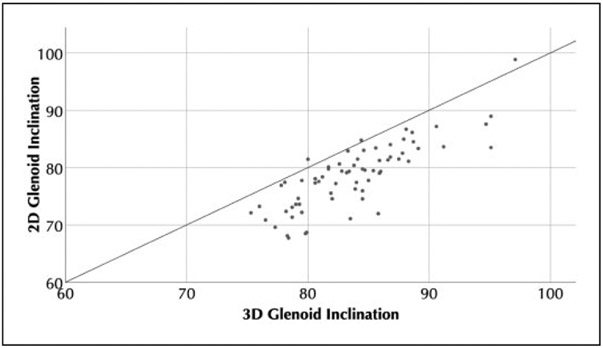
Scatter plot showing 3D glenoid inclination (*β*) versus 2D glenoid inclination (*β*).

In total, the 68 cadaver CT scans included 36 females and 32 males, with 35 left, and 33 right scapulae. The mean ± standard deviation age was 60 ± 10 years (range, 26–73 years). The gold-standard 3D *β* angle was 83 ± 5° (range, 72°–98°). On neutral plain radiographs, the average 2D *β* angle was 80 ± 6° (range, 66°–99°). For reference, a *β* angle of 80° corresponds to 10° of superior inclination. Paired Student’s *t* tests demonstrated significant differences between each measurement methodology (*P* < .001 for 2D *β* angle vs 3D *β* angle, Figures 4 and 5). Bland-Altman plots did not demonstrate any trend between measurement size and measurement difference. Bland-Altman plots show a mean difference between 2D *β* angle and 3D *β* angle of 5°, and that in 95% of cases, the difference between the 3D *β* angle and the 2D *β* angle was between −1° and 12°.

### Reliability of Radiographic Measurement of GI

Both the neutral 2D radiographic *β* angle and the 3D *β* measurements demonstrated ICCs above our 0.75 threshold of acceptability (Table 1). However, the reliability of *β* angle measurement was highly dependent upon viewing angle (Table 2). Specifically, ICCs were only above 0.75 for extended views, the 10° anteversion view, and the combined 15° anteversion and 15° extension view, all other views had ICCs of <0.75. Interrater ICCs for each radiographic viewing angle varied widely from 0.24 (95% confidence intervals −0.07 to 0.67) for the 15° retroverted and 15° flexed position to 0.88 (0.63–0.97) in the 15° extended position. Intrarater ICCs were >0.90 for the neutral 2D radiographic viewing angle, the 3D inclination measurement (Table 3).

## Discussion

The purposes of this study were to determine (1) whether GI could be accurately measured on plain radiographs as compared to a gold-standard 3D measure and (2) whether GI could be reliably measured on plain radiographs. Our hypotheses were partially supported. In the neutral view, the 2D *β* angle underestimated the 3D *β* angle by an average of 5° and are thus inaccurate. GI reliability was dependent upon viewing angle. These results suggest that in clinical decisions and future research regarding GI 3D imaging is necessary to accurately and reliably measure inclination.

Our study has several limitations. First, we utilized CT scans from shoulders grossly free of osteoarthritis or rotator cuff tears. Medical histories were not available for these cadavers and thus other shoulder pathologies or more subtle presentations of osteoarthritis or rotator cuff pathology may have been present. Second, our results may or may not be generalizable to patients with osteoarthritis or rotator cuff tears, and both pathologies may be influenced by GI.^1-5^ Because a large population of cadaver scapulae with osteoarthritis and/or rotator cuff tears are not available to perform this type of controlled laboratory study, this limitation is inherent to this type of research. Third, because of the strict imaging criteria of the commercially available software, the DICOM files of our database could not be analyzed by this software and were analyzed manually to create gold standard measurements. However, this methodology has been previously shown to be accurate and reliable.^14,30^ Fourth, one of the individuals who measured the *β* angle also created the DRRs. However, the randomized and blinded study design and the involvement of other observers mitigates this potential for bias. Fifth, the sample size for the multiple 2D viewing perspectives was limited to 10 subjects and may thus be underpowered. This power issue is illustrated by the difference in ICCs between the full 68 cadaver sample (Table 1, 0.79) and the 10 cadaver sample (Table 2, 0.65) from the neutral viewing perspective. Sixth, our study used DRRs instead of radiographs. Theoretically, in a DRR, a 3D view is “flattened” into a 2D image, while in an actual radiograph there are parallax effects from the use of a single x-ray emission source. However, the use of DRRs has been previously validated for making measures on simulated plain radiographs in the pelvis, which has a greater volar to dorsal distance than the scapula and is therefore more affected by this effect.^21^

Few prior studies have assessed the accuracy of the *β* angle. Our results are similar to those of Daggett et al., who demonstrated a significant mean difference of 3° between radiographic *β* angle measurements and their gold standard.^12^ These authors used an automated method for inclination measurement,^13,30^ which unfortunately could not be used in our study because this software would not analyze the available imaging data available. They are in contrast to those of Van Haver et al., although their study of the *β* angle was in patients after shoulder arthroplasty where the glenoid may be more easily definable radiographically.^13^ Based upon our results, researchers should consider adding 5° to these measures so they will approximate the 3D gold-standard measures. Otherwise plain radiographs may not be suitable for measurement of inclination. Within our study, inclination as measured by each methodology varied over 30°, which is similar to a prior study.^11^ Although the importance of this variance remains unknown, abnormalities in GI may be linked to rotator cuff tears and osteoarthritis.^1-5^ These prior studies have suggested that differences of 2° to 3° may be important in this risk differential, and thus, in the authors opinion, the 5° measurement inaccuracy described in our study should be considered clinically important. The precise source of this measurement inaccuracy is unclear, but it may be related to consistent difficulty precisely defining the angle of the supraspinatus fossa on plain radiographs.

Within our study, *β* angle was measured reliably on neutral radiographs and with the 3D methodology, but it could not reliably be measured on many other radiographic viewing angles. No prior studies have examined whether inclination measurement reliability changes with viewing angle and thus our conclusions are in contrast to some prior studies, who have suggested that inclination can be accurately and reliably measured on plain radiographs.^11,13^ However, our results are similar to those of Daggett et al., who demonstrated an interrater ICC for the *β* angle of 0.70, below our 0.75 level of acceptability.^12^ They are also similar to Zwingenberger et al., who demonstrated unacceptably high intra- and interobserver variance for the *β* angle in a controlled laboratory study.^31^ Our intrarater reliability was consistently above 0.90 regardless of measurement method, which may be due to differences in landmarks selected for measurement methods. Our results are in contrast to those of Van Haver et al., although these measurements were made for patients after shoulder arthroplasty, where the glenoid is more easily visualized radiographically.^13^ Similar to our study, in their original description of the *β* angle, Maurer et al. also demonstrated the *β* angle to be dependent upon viewing angle.^11^ In their study, some viewing angles were associated with a >20% difference from the neutral plane. This translates to a 15° difference in *β* between the angle measured and the angle that would be measured in the neutral viewing plane. Within our study, differences of a similar magnitude were observed—for instance, for observer 1, the 30° flexion view angle was associated with an 8° mean difference from the neutral viewing angle (Table 2). This could have significant clinical implications if the degree of inclination correction or choice of implant hardware is directly impacted by the measured values. Unfortunately, it is currently unknown what minimum difference in *β* will impart a significant change in clinical or biomechanical outcomes of treatment. Our data describe the variation across many viewing perspectives in a controlled setting and may be used in the future to determine which views are acceptable once a clinical threshold is defined. Our results suggest that the 3D inclination measure has excellent inter- and intrarater reliability. Our interrater ICC for the 3D inclination measure was 0.83, which is above the threshold of acceptability.^27^ This result is similar to those of Daggett et al.,^12^ Chalmers et al.^14^ and De Wilde et al.,^32^ all of whom demonstrated that inclination measurements could be reliably made on CT images using a similar technique. Because clinical radiographs exist in a wide variety of viewing angle orientations, the dependence of reliability upon viewing angle suggests that clinically this measurement may not be reliable unless radiographs are collected using a controlled perspective technique.

## Conclusion

Two-dimensional radiographic GI measurement is not accurate, as it underestimates the 3D value by an average of 5° when compared to the gold-standard 3D measurement. GI 2D measurement reliability varies with viewing angle on plain radiographs and thus to accurately and reliably measure inclination 3D imaging is necessary.

## Figures and Tables

**Table 1.** Interrater ICCs for Each Measurement Method Across N = 68 Cadaver Scapulae.

3D CT	ICC
2D radiographic *β* angle	0.79 (0.41–0.9)
3D CT *β* angle	0.83 (0.6–0.92)

Abbreviations: CI, confidence interval; CT, computed tomography; ICC, intraclass correlation coefficients.

ICC (95% CI).

**Table 2.** Interrater ICCs for Each 2D Viewing Perspective in N = 10 Scapulae With Multiple Viewing Perspectives in Each.

Angle	ICC	Observer 1	Observer 2	Observer 3
Neutral	0.65 (0.2 to 0.89)	76.7 (4.4)	80.1 (3.8)	80 (3.4)
5° extension	0.74 (0.34 to 0.93)	77.2 (2.9)	80 (4.1)	79 (3.9)
10° extension	0.8 (0.49 to 0.94)	77 (2.9)	79.1 (4.6)	78 (3.8)
15° extension	0.88 (0.63 to 0.97)	76.2 (4.3)	78.2 (5.1)	77.1 (3.7)
30° extension	0.82 (0.5 to 0.95)	72.2 (5.4)	75.4 (6.1)	73.9 (4.9)
5° flexion	0.55 (0.17 to 0.84)	80.3 (3)	81.5 (3.8)	80.7 (3)
10° flexion	0.69 (0.27 to 0.91)	80.7 (3.2)	83.2 (2.7)	82.7 (3.2)
15° flexion	0.53 (0.15 to 0.83)	82.6 (2.5)	84 (3.9)	82.9 (3.7)
30° flexion	0.26 (−0.13 to 0.7)	84.4 (3.3)	84.9 (5.9)	85.6 (5.1)
5° anteversion	0.47 (0.1 to 0.8)	77.9 (3.5)	79.7 (3.1)	79.8 (3.3)
10° anteversion	0.77 (0.5 to 0.93)	77.8 (2.9)	78.8 (3.7)	79 (3.5)
15° anteversion	0.6 (0.24 to 0.86)	77.6 (4.1)	79.2 (4.4)	78 (3.5)
30° anteversion	0.64 (0.29 to 0.88)	75.3 (6.3)	77.8 (5)	74.9 (3.8)
5° retroversion	0.64 (0.27 to 0.88)	79.8 (2.3)	82.1 (3.1)	81.2 (3.3)
10° retroversion	0.37 (−0.02 to 0.76)	80.3 (2.5)	81.4 (2.9)	80.9 (3.3)
15° retroversion	0.58 (0.2 to 0.86)	79.3 (2.7)	81.3 (3.3)	81.4 (3.3)
30° retroversion	0.47 (0.04 to 0.81)	77.5 (4.1)	82.5 (2.6)	81.5 (3.8)
15° anteversion and 15° extension	0.81 (0.51 to 0.95)	71.5 (5.6)	74.1 (6)	73.4 (4.6)
15° anteversion and 15° flexion	0.45 (0.07 to 0.81)	83 (1.8)	83.5 (2)	81.8 (3.9)
15° retroversion and 15° extension	0.65 (0.22 to 0.9)	76.2 (5.3)	80.1 (3.9)	79 (4.2)
15° retroversion and15° flexion	0.24 (−0.07 to 0.67)	80.4 (2.8)	84.5 (3.5)	82.7 (5)

Abbreviations: CI, confidence interval; ICC, intraclass correlation coefficients.

ICC (95% CI). In addition, mean (standard deviation) measures for each observer are shown.

**Table 3.** Intrarater ICC for Each Measurement Methodology Across N = 68 Cadaver Scapulae.

Measurement Method	ICC
2D radiographic *β* angle	0.91 (0.85–0.94)
3D CT *β* angle	0.99 (0.98–0.99)

Abbreviations: CI, confidence interval; ICC, intraclass correlation coefficients.

ICC (95% CI).
